# White Matter Injury after Intracerebral Hemorrhage: Pathophysiology and Therapeutic Strategies

**DOI:** 10.3389/fnhum.2017.00422

**Published:** 2017-08-25

**Authors:** Chuanyuan Tao, Xin Hu, Hao Li, Chao You

**Affiliations:** Stroke Clinical Research Unit, Department of Neurosurgery, West China Hospital, Sichuan University Chengdu, China

**Keywords:** intracerebral hemorrhage, white matter injury, pathophysiology, axonal damage, demyelination, treatment

## Abstract

Intracerebral hemorrhage (ICH) accounts for 10%–30% of all types of stroke. Bleeding within the brain parenchyma causes gray matter (GM) destruction as well as proximal or distal white matter (WM) injury (WMI) due to complex pathophysiological mechanisms. Because WM has a distinct cellular architecture, blood supply pattern and corresponding function, and its response to stroke may vary from that of GM, a better understanding of the characteristics of WMI following ICH is essential and may shed new light on treatment options. Current evidence using histological, radiological and chemical biomarkers clearly confirms the spatio-temporal distribution of WMI post- ICH. Although certain types of pathological damage such as inflammatory, oxidative and neuro-excitotoxic injury to WM have been identified, the exact molecular mechanisms remain unclear. In this review article, we briefly describe the constitution and physiological function of brain WM, summarize evidence regarding WMI, and focus on the underlying pathophysiological mechanisms and therapeutic strategies.

## Introduction

Intracerebral hemorrhage (ICH) accounts for approximately 10%–15% of all strokes in Western countries and 20%–30% of strokes in Asia and has a high mortality and poor functional outcome. Less than 30% of patients are independent 1 year after the stroke onset (Samarasekera et al., 2015). ICH leads to both gray matter (GM) and white matter (WM) injury (WMI). Indeed, the typical clinical syndromes, such as contralateral hemiplegia due to injury to the corticospinal tracts (CSTs) and corticonuclear tracts, hemidysesthesia due to injury to the central thalamic radiations and hemianopia due to damage to the optic radiation after deep basal ganglia ICH are the main sequelae resulting from WMI (Chung et al., 2000; Qureshi et al., 2001). Additionally, the cognitive dysfunction following striatal ICH may predominantly reflect injury to adjacent WM pathways rather than damage to the putamen or caudate (Smith and Venegas-Torres, 2014). Therefore, WMI is a great contributor to the neurological deficits after ICH, but experimental and clinical studies focus more on GM damage than WMI, which may be partially responsible for the failure of treatments with massive neuroprotectants targeting degenerating neuronal cells (Wasserman and Schlichter, 2008). Fortunately, the importance of ICH-induced WMI has been increasingly recognized and WMI has been recommended as a priority for basic and clinical stroke research (NINDS ICH Workshop Participants, 2005). However, current studies remain limited in their understanding of WMI in ICH. A comprehensive review of the pertinent literature that discusses the pathophysiological mechanisms underlying WMI, potential therapeutic targets and novel treatment modalities is essential.

## WM Distribution in The Brain

The central nervous system consists of the following two types of brain tissue: GM and WM. In humans, nearly half of the brain is composed of GM and half is composed of WM. The GM is mainly composed of neuronal cell bodies and unmyelinated axons while the WM contains myelinated axon tracts and, supporting glia cells, including oligodendrocytes, astrocytes and microglia. Axons are tightly wrapped by multiple myelin sheaths that are produced by mature oligodendrocytes, and the integrity of the myelin sheath is vital for accurate and high speed nerve signal conduction. According to the connectivity and functionality, WM can be further divided into projection tracts, commissural tracts and association tracts (Gerrish et al., 2014). Projection tracts transmit nerve signals from the cortex to other regions of the brain, including the CST and optical and thalamocortical radiations. The commissural tracts allow the left and right cerebral hemispheres to communicate. The association tracts connect one cortical lobe with another within the ipsilateral hemisphere. WM accounts for approximately half of the forebrain volume (Ge et al., 2002) and may account for a greater proportion in the hindbrain, particularly in the brainstem, in humans.

## The Function of WM

WM is believed to be passive tissue that acts as a relay and coordinates communication among different regions of GM. However, WM has recently been shown to affect neuron functioning in learning and cognitive processes in the absence of pathology (Wang and Young, 2014). For example, the number of hours a professional musician practices is correlated with structural changes in WM (Ullén, 2009), and the volume and functional connectivity of the WM tracts that link the cortical regions that are vital for reading are increased when learning to read (Carreiras et al., 2009). Therefore, WM may have more diverse functions than is currently known. Under many pathological conditions, WMI can cause sensorimotor impairment, cognitive dysfunction, psychiatric disorders, gait disturbance, disequilibrium, urinary incontinence and pain, thus contributing to the development of critical neurological impairments (Schmahmann et al., 2008).

## Histological Evidence of WMI after ICH

Axonal injury and demyelination were first described in a collagenase-induced ICH rat model by Wasserman and Schlichter (2008) using immunohistochemical staining. Anti-bodies against myelin basic protein (MBP), degraded MBP (dMBP) and amyloid precursor protein (APP) were used to detect intact myelin, demyelination and axonal injury, respectively. The authors found obvious demyelination and axonal damage at the edge of the hematoma within 3 days, and the axonal injury progressively extended to the surrounding parenchyma over time, but no dMBP or APP accumulation was detected outside of the hematoma at a later time point of 28 days. These results are consistent with studies by Moxon-Emre and Schlichter (2011) and Zou et al. (2017) who discovered that APP nearly disappeared after 7 days using a blood infusion rat model. However, the demyelination detected using Luxol fast blue staining can persist for up to 2 months, suggesting that the WMI is long-lasting following ICH (Liu et al., 2010; Ni et al., 2015). Moreover, we further found that the significant demyelination and axonal damage 3 days post-ICH were highly associated with brain edema and neurologic dysfunction using an ICH rat model in which autologous blood was infused into the pons, indicating that WMI plays a vital role in neurologic impairment (Tao et al., 2016). Although these histological studies were all performed in rats, which have low levels of WM, the WMI was remarkable during the acute stage and may persist for a longer period of time, which is likely related to the residual disability observed in most clinical patients. Further studies in animals that are rich in WM may provide evidence of more widespread WMI caused by ICH.

Oligodendrocytes, which are the most important cellular components of WM, are vulnerable to hemorrhagic insults (Zhuo et al., 2016). Oligodendrocyte death has been shown to be accompanied by oligodendrocyte precursor cell proliferation during the acute period in the peri-hematoma WM (Sahinkaya et al., 2014; Joseph et al., 2016; Tao et al., 2016; Zhuo et al., 2016). Regarding the specific pathogens, iron released from hemoglobin breakdown is commonly believed to damage oligodendrocytes because an increase in intracellular Fe^2+^ is toxic to oligodendrocytes, and iron chelator inhibits oxidative toxicity in oligodendrocytes *in vitro* (Masuda et al., 2007). Other mechanisms underlying oligodendrocyte apoptosis involve endoplasmic reticulum and mitochondrial pathways, and oligodendrocyte apoptosis was observed to be accompanied by an upregulated expression of caspase-12 and enhanced release of cytochrome c (Zhuo et al., 2016). Specific inhibitors blocking these apoptotic pathways are expected to reduce apoptosis in oligodendrocytes.

In summary, WMI that is characterized by demyelination, axonal damage and loss of oligodendrocytes frequently occurs within 3 days of the onset of ICH, implying that a relatively wide therapeutic time window may exist in ICH which is advantageous for the design of potential treatments.

## Detection of WMI after ICH Using Advanced MRI Techniques

Histological techniques are rarely applicable as diagnostic tool in clinical practice because tissue specimens are difficult to obtain. Hence, noninvasive advanced MRI is more useful for the detection of WMI with a greater translational potential. Diffusion tensor imaging (DTI) has evolved considerably over the previous decade and can currently detect the magnitude and directionality of water molecules in WM with a higher sensitivity during WMI monitoring than conventional MRI. By calculating certain technical parameters such as the fractional anisotropy (FA), mean diffusivity (MD), axial diffusivity (λ_//_) and radial diffusivity (λ_⊥_), DTI can assess the integrity and connectivity of WM tracts, reconstruct the three-dimensional distribution of WM pathways in the brain, and help determine the WM microstructural pathophysiology. For instance, a decreased FA, a decreased λ_//_, and an increased λ_⊥_ reflect general WMI, axonal damage and myelin injury, respectively (Song et al., 2003; Fox et al., 2011).

The CST, which consists of the internal capsule, the cerebral peduncle and pyramid tract, is one of the most prominent pathways related to motor functional outcome after ICH (Liang et al., 2013; Venkatasubramanian et al., 2013; Cheng et al., 2015). The application of DTI for detecting CST injury after hemorrhagic stroke has been recently reviewed with a particular focus on the predictive value of DTI in ICH (Chaudhary et al., 2015). In general, FA measurements in the affected CST, particularly in the cerebral peduncles, may assist in predicting the motor function outcome within 6 months and the selection of patients who may benefit from surgical hematoma evacuation. A recent meta-analysis further confirmed these results by demonstrating a strong correlation between the FA value and upper extremity motor recovery in ICH patients (Kumar et al., 2016).

A routine diffusion-weighted imaging (DWI) sequence is sensitive to altered tissue water content and can be used to detect Wallerian degeneration (WD) after ICH. CST-WD changes have been observed 1 week following ICH as reduced diffusion on DWI that progresses rostrocaudally along the CST (Venkatasubramanian et al., 2013). Moreover, the presence of CST-WD is associated with poor motor and functional recovery after ICH, particularly in the deep basal ganglia (Karibe et al., 2000; Venkatasubramanian et al., 2013). Compared to DTI, DWI is readily available using routine clinical MRIs and can be easily interpreted by clinicians without the need for sophisticated quantification of certain parameters, such as FA, MD, λ_//_ and λ_⊥_, that are required for DTI.

Overall, advanced MRI techniques, including DTI and DWI, can serve as radiological markers of WMI in ICH patients for prognostication, which is important for the treatment and rehabilitation plan. More widespread application is encouraged, particularly in recovered patients with a good modified Rankin Scale (mRS) grade who continue to suffer from neuropsychological deficits, because advanced MRI techniques may also provide more diagnostic information regarding cognitive function than conventional CT and MRI.

## Biomarkers of WMI after ICH

WMI causes disintegration of axons and myelin, facilitating the outflow of intracellular contents into the extracellular space, their diffusion into the cerebrospinal fluid (CSF), and even their entry into the blood stream through a disrupted blood-brain barrier (BBB). Neurofilaments are the major components of WM, constituting the axonal cytoskeleton. Neurofilaments are mainly composed of heavy (NF-H) and light (NF-L) chains (Al-Chalabi and Miller, 2003). Neurofilament subunits are accepted as biomarkers of axonal injury in traumatic brain injury (TBI; Gatson et al., 2014), ischemic stroke (IS; Sellner et al., 2011; Tuor et al., 2014) and other neurodegenerative disorders (Lu et al., 2015). The elevated levels of these biomarkers in ICH have recently attracted interest regarding their diagnostic and predictive values. The significant elevation in phosphorylated NF-H both in the CSF and plasma was detected in ICH patients, providing evidence of primary and secondary axonal injury (Petzold et al., 2005; Sellner et al., 2011; Cai et al., 2013). For example, the plasma level of phosphorylated NF-H up on admission was identified as a reliable and independent marker that is predictive of patients at risk for early neurological deterioration and 6-month poor clinical outcomes in patients with ICH (Cai et al., 2013).

Tau is another cytoskeleton protein that is highly concentrated in axons. Increased concentrations of tau in the CSF have been suggested to reflect axonal degeneration. Serum tau protein was elevated and identified as a candidate marker of axonal injury in acute IS (Bitsch et al., 2002) and subarachnoid hemorrhage (SAH; Hu et al., 2012). The increased concentrations of tau in the serum have also been found to be associated with a high mortality and poor functional outcome at 3 months in ICH patients (Zanier et al., 2013). In particular, a baseline serum tau concentration >91.4 pg/mL can predict a poor outcome at 3-month with 83.6% sensitivity and 75.8% specificity. This predictive ability is comparable to that of the NIHSS score. However, dynamic samplings are required for the determination of whether serial tau measurements add more accurate prognostic information and define the optimal time points for such measurements.

Analyses of the biomarkers of demyelination in ICH have rarely been reported. Most studies have concentrated on MBP in multiple sclerosis (MS). MBP-like materials or fragments can be detected in the CSF in MS patients, and increase rapidly during acute exacerbation and subside over the following 4–6 weeks. Similarly, increases in the CSF and serum levels of MBP have been reported following TBI of various severities, and serum concentrations of MBP can predict the eventual GOS score (Berger et al., 2010; Su et al., 2012). Biomarkers, including MBP, used for an acute diagnosis and management of stroke have been recently reviewed (Jauch et al., 2006; Glushakova et al., 2016). One early study has shown increased levels of MBP in the CSF of patients with IS and ICH (Strand et al., 1984). MBP was found to be elevated in CSF samples from patients with ICH collected at times ranging from 0 days to 3 days after stroke and further increased on average by day 5 (Strand et al., 1984). The MBP levels have been confirmed to be correlated to the stroke severity at baseline, and higher values were predictive of a poor short-term prognosis; however, data regarding the direct relationship between the concentrations of MBP and the severity of the WMI remain lacking.

These bedside, relatively cheap and convenient molecular biomarkers of WMI could play an increasingly important role in prognostic evaluations and, treatment efficacy assessments as we shift our research focus from animal models to human patients. However, most of the abovementioned biomarkers were evaluated retrospectively only at a single center with a small sample size and are not currently used in the clinic. The sensitivity and specificity of these biomarkers must be validated in large prospective studies before clinical translation. The identification of other biomarkers based on future finding regarding novel distinct pathological mechanisms are anticipated. Because the biomarkers in the blood are convenient to obtain but lack specificity and are easily affected by systemic conditions and biomarkers in the CSF have a relatively high specificity but lack accessibility, identifying novel biomarkers in the blood with a high specificity has become quite urgent.

## Pathophysiological Mechanisms of WMI

**Figure 1:**
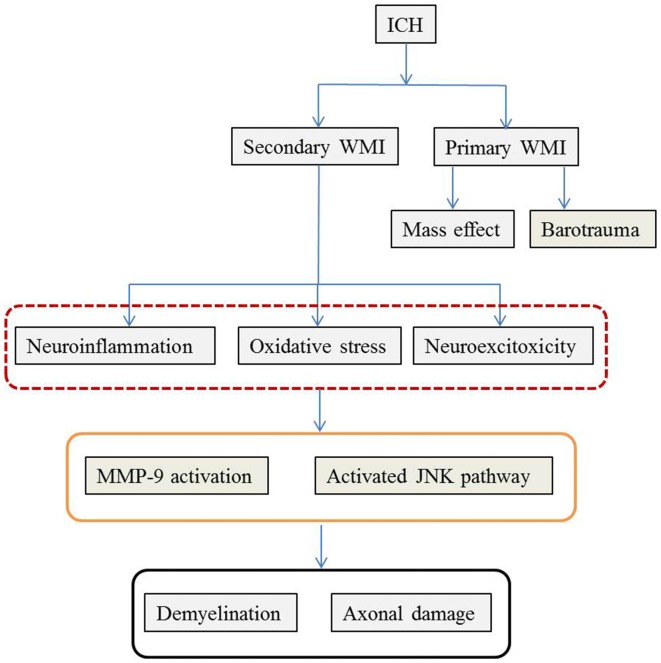
The possible pathophysiological mechanisms of WMI following ICH. The pathophysiology of WMI is summarized according to the available literature. ICH causes WMI via primary and secondary injury. Mass effect during hematoma formation and mechanical barotrauma mechanism are responsible for the primary WMI. Later on, secondary cascades including neuroinflammation characterized by inflammatory cell infiltration and cytokines and chemokines release, oxidative stress induced by hemoglobin and its metabolites, and glutamate-mediated neuro-excitotoxicity further deteriorate WMI. Although the detailed molecular mechanisms remain unclear, MMP-9 and JNK mediated events play important roles. The final consequences are disintegration of WM with demyelination and axonal damage. ICH, intracerebral hemorrhage; WMI, white matter injury; MMP-9, matrix metalloproteinase 9; JNK, Jun N-terminal kinase.

As previously mentioned, ICH-induced brain injury involves complex pathophysiological mechanisms that destroy both GM and WM. Similar to TBI, primary and secondary brain damage are observed following ICH-induced WMI. The mass effect and barotrauma during hematoma formation exert a destructive effect immediately after ICH whereas neuroinflammation, oxidative stress and excitotoxicity play a critical role in the subsequent secondary WMI. The pathophysiological mechanisms are summarized in Figure 1.

### Primary Biomechanical Mechanisms

**Figure 2:**
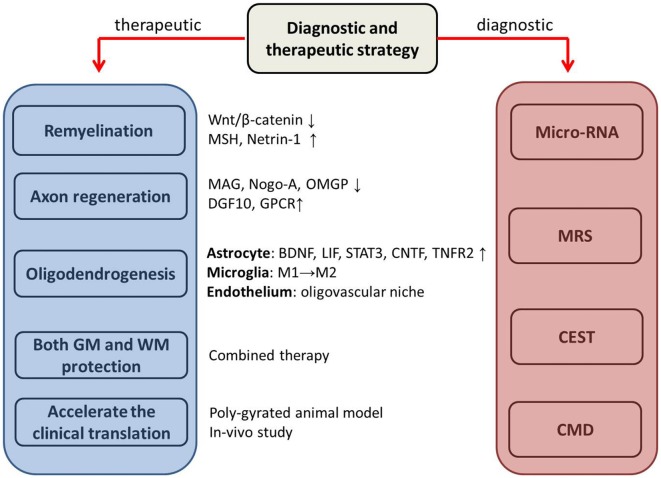
The potential therapeutic and diagnostic strategies. MSH, morphogen Sonic hedgehog; MAG****, myelin-associated glycoprotein; OMGP, oligodendrocyte myelin glycoprotein; DGF10, differentiation factor 10; GPCR, cell-type-specific G protein-coupled receptor; BDNF, brain-derived neurotrophic factor; LIF, leukemia inhibitory factor-like protein; STAT 3, signal transducer and activator of transcription 3; CNTF, ciliary neurotrophic factor; TNFR2, tumor necrosis factor receptor 2; GM, gray matter; WM, white matter; MRS, Magnetic resonance spectroscopy; CEST, Chemical Exchange Saturation Transfer; CMD, cerebral microdialysis. ↓ downregulate; ↑ upregulate; M1→M2, switch from pro-inflammatory M1 and the late anti-inflammatory M2.

Following ICH, the mass effect during hematoma formation can physically distend, distort and finally disrupt loose WM fibers. Those nerve fibers within the hematoma cannot recover, while the fibers surrounding the peri-hematoma show damage of various degrees of severity depending on the bleeding speed and hematoma size. Alternatively, to date, the biomechanical mechanism of barotrauma was considered similar in ICH and TBI because many similar pathophysiological changes between TBI and ICH have been observed, such as bilateral diffuse brain injury and brain atrophy due to an unilateral brain lesion (Powers, 2010; Kummer et al., 2015). In contrast to TBI, in which the barotrauma arises externally from a blow to the head, the barotrauma in ICH originates from the fluid percussion waves due to the sudden expansion of the hematoma under arterial pressure in the cranium and immediately propagates through the intracranial contents (Powers, 2010), resulting in distal axotomy and demyelination. (Figure 2). The mechanism of the barotrauma may partially explain the global WMI observed during the acute stage after regional ICH.

### Neuroinflammation

Inflammation is a major contributor to secondary WMI. Inflammation progresses in response to various stimuli produced after hemorrhage via the activation of microglia, peripheral inflammatory infiltration and the production of cytokines and chemokines. In addition, local microglial activation and peripheral leucocyte infiltration mutually interact to propagate inflammatory injury. The inflammatory reaction to ICH within WM has been demonstrated; for instance, nuclear factor kappa B (NF-κB) is rapidly activated, the pro-inflammatory cytokine genes are upregulated in the perihematomal WM (Wagner et al., 2006) and CD47, which is a transmembrane protein that regulates the entry of leukocytes into the brain, is elevated in the oligodendrocytes and microglia in the WM after experimental ICH (Zhou et al., 2014). Inhibiting inflammation can reduce WMI, providing further evidence of inflammation-induced WMI. For instance, inhibiting one of the pro-inflammatory factors, i.e., EP1R, which is expressed primarily in axons, could attenuate WMI, reduce brain atrophy and improve functional outcomes in an ICH animal model (Zhao et al., 2015). Furthermore, neutrophil depletion before ICH induction in the rat striatum reduced the infiltration of activated microglia in the peri-hematoma WM tracts and decreased myelin fragmentation and axon damage (Moxon-Emre and Schlichter, 2011).

Although the exact mechanisms of the inflammation-mediated WMI remain elusive, matrix metallopeptidase 9 (MMP-9) is considered to play a key role during this process. MMP-9 is produced by leukocytes, astrocytes and oligodendrocytes, and the MMP-9 promoter region contains activator protein-1 and NF-κB, both of which respond to inflammatory stimuli and specifically attack the basal lamina through the fibronectin binding domain (Seo et al., 2013; Vafadari et al., 2016). MMP-9 has been reported to be activated after ICH with consequential WMI, and many inflammatory mechanisms underlying WMI are also associated with the activation of MMP-9. For instance, EP1R was found to have a toxic effect on the WM surrounding the hematoma via the Src kinases–MMP-9 signaling pathway following ICH (Zhao et al., 2015); neutrophil depletion leads to attenuated myelin fragmentation and axon damage with reduced MMP-9, indicating that MMP-9 produced by infiltrated neutrophils may contribute to WMI (Moxon-Emre and Schlichter, 2011). Another inflammatory signaling axis recently identified using an ICH model, i.e., iron—the receptor for advanced glycation end-products (RAGE)—NF-κB, is associated with the BBB and WMI via the activation of MMP-9 (Yang F. et al., 2015). However, in contrast to its deleterious role during the early period, MMP-9 secreted by oligodendrocytes can promote WM remodeling by accelerating angiogenesis that is mediated by an analogous oligovascular niche during the recovery stage after lysophosphatidylcholine-induced WMI in mice (Pham et al., 2012). It is unclear whether the late beneficial effect of MMP-9 exists and contributes to WM repair following ICH. If this hypothesis is confirmed in the future, treatments targeting MMP-9 should avoid the complete inhibition of MMP-9 during all stages.

### Oxidative Stress

WM is vulnerable to oxidative stress induced by ischemic or hemorrhagic events (Hall et al., 2000; Wang Y. et al., 2016). Wagner et al. (2002, 2005), evaluated the oxidative injury in WM by measuring the formation of protein carbonyl and gene expression of HO-1 after infusing either plasma or whole blood into the porcine frontal WM and concluded that not only whole blood but also its plasma components are capable of rapidly inducing oxidative stress in WM.

Erythrocyte lysis followed by the release of hemoglobin and its metabolites occurs several days after ICH (Cao et al., 2016). Heme, which is the major product of hemoglobin, can be degraded into iron, carbon monoxide and biliverdin by heme oxygenase (HO; Hu et al., 2016). Iron overload in the brain subsequently generates abundant reactive oxygen species via the Fenton reaction (Hu et al., 2016), resulting in WMI. Iron oxidation has been shown to cause greater oxidative stress in the WM than GM *in vitro* (Hall et al., 2000). An intrastriatal injection of FeCl_3_ in rats leads to remarkable axonal damage and demyelination (Zou et al., 2017), while iron chelators, such as minocycline (Zou et al., 2017) and deferoxamine (Gu et al., 2009; Xie et al., 2014; Ni et al., 2015), decrease iron accumulation, attenuate WMI, and improve neurological function in rodents and piglets. Furthermore, another metabolic by-product of hemoglobin, i.e., bilirubin, has been reported to cause structural and functional damage in the WM *in vitro* and *in vivo* through its oxidation products (Lakovic et al., 2014).

Iron-induced oxidative stress and inflammation injury can overlap to further aggravate WMI through the iron dependent c-Jun N-terminal kinase (JNK) pathway. JNK is a member of the mitogen-activated protein kinase (MAPK) superfamily, which has already been widely investigated due to its active actions in response to various stimuli, such as the exposure to inflammatory cytokines (Kumar et al., 2015). JNK is activated in ICH animal models primarily by iron, and iron chelator can reduce the free iron contents in the CSF, suppress JNK activation, reduce WMI and improve neurological deficits (Yatsushige et al., 2007; Wan et al., 2009; Ni et al., 2015; Chen et al., 2016; Zou et al., 2017). Furthermore, the inhibition of JNK could deactivate inflammation, attenuate brain edema and improve functional outcome after ICH (Ohnishi et al., 2007; Michel-Monigadon et al., 2010). Interestingly, similarly to MMP-9, the JNK pathways have been found to promote axonal regeneration after cytoskeletal disruption (Quintanilla et al., 2013; Valakh et al., 2015). Thus, iron is an ideal potential candidate for the treatment of WMI post-ICH due to its dual toxic roles in oxidative stress and inflammation.

### Glutamate-Mediated Excitotoxicity

Preclinical studies have revealed that the level of glutamate is up to eight times higher than the baseline value during the early period of ICH due to the influx of glutamate from the hematoma or through a disrupted BBB from circulation (Qureshi et al., 2003), and these values correlate with the brain edema and BBB disruption (Wu et al., 2013b). Moreover, the perihematomal glutamate level is associated with the postoperative outcome in ICH patients (Wu et al., 2013a). For hemiplegic patients with basal ganglia hematoma affecting the internal capsule, earlier minimally invasive surgery is accompanied by a lower level of peri-hematoma glutamate and a better prognosis, indicating that glutamate-related WMI may play a role in the functional outcome, and the early attenuation of glutamate-induced WMI may enhance recovery (Wu et al., 2013a). The increased glutamate activates glutamate receptors in oligodendrocytes and axons with intracellular Ca^2+^ overload, leading to oligodendrocyte death, demyelination and axon injury in MS, TBI, spinal cord injury and cerebral ischemia (Park et al., 2004; Matute et al., 2007; Matute, 2011; Zhang et al., 2013). Similarly, glutamate-mediated excitotoxicity is believed to play a detrimental role in WMI following ICH, although pertinent studies are lacking.

## Therapeutic Prospective (Figure 2)

### Accelerating Oligodendrogenesis, Remyelination and Axon Regeneration

The molecular mechanisms of WMI after ICH are currently unclear. More thorough research regarding studies exploring the potential mechanisms underlying WMI are required to identify novel treatment targets. Oligodendrocyte differentiation and remyelination are vital for WM repair. The expression and function of either their negative regulators, including the Wnt/β-catenin signaling pathway (Dai et al., 2014; Lee et al., 2015a,b) and myelin-associated inhibitory factors, or positive regulators, such as the morphogen Sonic hedgehog (MSH; Ferent et al., 2013) and trophic factors such as Netrin-1 (He et al., 2013; Tepavčević et al., 2014), should be investigated in the context of ICH in future studies.

Accelerating axon regeneration is another option for restoring the integrity of WM. Myelin-associated inhibitors, such as myelin-associated glycoprotein (MAG), Nogo-A, and oligodendrocyte myelin glycoprotein (OMGP), can lead to growth cone collapse and inhibit axon regeneration (Muramatsu and Yamashita, 2014). However, while a wealth of new data regarding activating axonal repair after IS and other neurological diseases are available, this knowledge has not yet been applied to ICH. For example, the induction of growth and differentiation factor 10 (GDF10) in peri-infarct neurons can produce axonal sprouting and enhance functional recovery (Li et al., 2015), and cell-type-specific G protein-coupled receptor (GPCR) signaling essential for axonal guidance and targeting during development was recently found to promote axon regeneration after CNS injury (Li et al., 2016). By targeting modulators that are directly related to the physiological processes of axonal regeneration, new treatment can be developed.

### Crosstalk between Oligodendrocytes and Other Cells

In addition to oligodendrocytes, other cell types, including astrocytes, microglia and endothelial cells, are contained in the WM, and their interaction is essential for the maintenance of the integrity of the WM. Crosstalk among these cell components is required for oligodendrogenesis and remyelination after WM damage (Pham et al., 2012; Itoh et al., 2015; Miyamoto et al., 2015; Rosenzweig and Carmichael, 2015). Therefore, an understanding of the signaling pathways involved in intracellular crosstalk will add new knowledge that will be helpful for alleviating WMI and accelerating WM restoration. Astrocytes support oligodendrocyte function. Several soluble factors secreted by astrocytes have been implicated in stimulating oligodendrocyte differentiation and enhancing myelination. *in vitro* and *in vivo* experiments have demonstrated that astrocyte-derived brain-derived neurotrophic factor (BDNF) supports oligodendrogenesis and regeneration after WM damage (Miyamoto et al., 2015). Furthermore, leukemia inhibitory factor-like protein (LIF), signal transducer and activator of transcription 3 (STAT 3), ciliary neurotrophic factor (CNTF) and tumor necrosis factor receptor 2 (TNFR2) promote oligodendrocyte survival and maturation (Patel et al., 2012; Fischer et al., 2014; Monteiro de Castro et al., 2015; Domingues et al., 2016). Microglia also play a crucial role in remyelination by driving the phagocytosis of myelin debris and oligodendrocyte differentiation during CNS disorders. After ICH, microglia can be polarized into two phenotypes, i.e., the early pro-inflammatory M1 and the late anti-inflammatory M2 (Zhang et al., 2017). The switch from M1 to M2 can enhance oligodendrocyte differentiation, and M2 cell polarization is essential for efficient remyelination via M2 cell–derived activin-A after demyelination (Miron et al., 2013). Additionally, endothelial-oligodendrocyte interactions may be important for WM repair after stroke (Arai and Lo, 2009; Pham et al., 2012). The “oligovascular niche” was recently proposed as a microenvironment between cerebral endothelial cells and the oligodendrocyte lineage in which these cells exchange soluble factors, such MMP-9, BDNF and vascular endothelial growth factor (VEGF), to maintain WM homeostasis (Miyamoto et al., 2014). Under stroke conditions, oligovascular coupling is interrupted, which may contribute to WMI (Arai and Lo, 2009). A deeper understanding of the mechanisms of endothelial-oligodendrocyte trophic coupling may lead to new therapeutic approaches for ICH.

### Protection of Both GM and WM

ICH damages both the GM and WM. It is hypothesized that the sole focus on neuronal protection is partially responsible for the failure of ICH treatments (NINDS ICH Workshop Participants, 2005). However, focusing only on WMI may also be fruitless. Therefore, treatments targeting both GM and WM are encouraged in the future. However, a single treatment is not easy to develop because there are significant distinctions in the cellular properties between the GM and WM. Therefore, developing agents with multiple pharmaceutic characteristics; combining therapeutic modalities, such as surgical hematoma evacuation, followed by drug therapy; or combining multiple agents with separate roles may be the management of choice.

### Translation from the Bench to the Clinic

Currently, to accelerate the translation from the bench to the clinic, several points should be noted. Regarding animal studies, rodents are the predominant species used in stroke research studies worldwide. However, the rodent WM comprises only 14% of the total brain tissue, whereas this proportion reaches 50% in humans (Zhang and Sejnowski, 2000; Rosenzweig and Carmichael, 2015). Other animal models, such as pigs, with poly-gyrated brains are needed, and nonhuman primates are even more ideal for assessing WMI. In addition, advanced MRI equipped with a multi-parameter measuring system is most advantageous for dynamically observing the integrity and microstructural changes in the WM throughout the whole period in living subjects and should be routinely incorporated into the evaluation of WMI after ICH. More importantly, more *in vivo* studies of WMI in patients are desperately needed because of the translational difficulty of the neuroprotection benefits observed in animal studies.

## Diagnostic and Prognostic Outlook

### microRNA (miRNA)

microRNA (miRNA) is an important posttranscriptional regulator of gene expression that has been implicated in the pathogenesis of many human diseases and has been suggested as a possible diagnostic and prognostic biomarker. Experimental studies showed a distinct miRNA profile after ICH, which was involved in inflammatory injury and apoptosis (Kim et al., 2014; Yang Z. et al., 2015; Yuan et al., 2015). Analyses of peripheral blood and hematoma samples from ICH patients further reveal that there are some specific miRNAs that are closely correlated to the enlargement of subsequent hematomas, WM edema and functional outcome (Zheng et al., 2012; Zhu et al., 2015; Wang J. et al., 2016; Wang M. D. et al., 2016). Furthermore, certain miRNAs have been regarded as candidate biomarker of WMI in certain neurodegenerative disorders (Gandhi, 2015; Kuswanto et al., 2015). For example, microRNA-137 is associated with the integrity of WM and neurocognitive functioning in patients with schizophrenia (Kuswanto et al., 2015). Therefore, because of their additional advantages of wide distribution, stability and relative ease of detection (Stylli et al., 2017), miRNAs may provide insight into the mechanisms of WMI and prognosis after ICH.

### Other Advanced MRI Techniques

Magnetic resonance spectroscopy (MRS) allows for the noninvasive measurement of *in vivo* metabolites in a predefined brain region of interest. The metabolic changes in N-acetylaspartate and total creatines in the peri-stroke area are correlated with the WM loss (Yassi et al., 2016), and their ratio is a useful indicator for the early diagnosis of WMI and cognitive impairment in cerebral ischemic patients (Xing et al., 2013). Since the concentrations of N-acetylaspartate, total choline and total creatines are a reflection of axonal health, cellular membrane integrity and energy metabolism, respectively (Acheson et al., 2014), their metabolic activities in the WM after ICH might represent the corresponding axonal damage and demyelination. This hypothesis is supported in patients with TBI (Sinson et al., 2001; Grossman et al., 2015). The MRS technique could be beneficial for detecting WMI early before morphological changes occur.

Although MRS-based techniques can detect changes in most cerebral metabolites, their spatio-temporal resolution is often insufficient for routine examinations of fast-evolving and heterogeneous acute stroke lesions (Sun et al., 2011). Chemical Exchange Saturation Transfer (CEST) has emerged as a novel MRI technique for tissue pH imaging and imaging of various metabolites and is well suited for molecular imaging studies (Liu et al., 2013). Among the techniques of CEST MRI, pH-weighted and Amide Proton Transfer (APT) MRI have been implicated in IS as a surrogate of lactate acidosis and altered tissue metabolism (Sun et al., 2011; Tietze et al., 2014). Although CEST MRI is in its infancy in the field of stroke research, with the development of novel sequence and post-processing methods, it is hopeful that a better understanding of the mechanism of WMI following ICH will be obtained by discovering the subtle metabolic changes.

Cerebral microdialysis (CMD) is a well-established laboratory tool that provides an on-line analysis of the brain biochemistry via a thin fenestrated dialysis catheter that is inserted into the brain parenchyma. In addition to the monitoring of energy metabolism, CMD can dynamically observe excitotoxicity, inflammation and oxidative stress by measuring the levels of glutamate, metalloproteinases, cytokines and 8-iso-PGF_2_, respectively (de Lima Oliveira et al., 2014). Moreover, axonal skeleton proteins, such as tau, can also be quantified using CMD (Magnoni et al., 2012). Hence, this technique has great potential in the study of the pathophysiology underlying the ICH-induced WMI.

### Summary

The importance of WMI in ICH is increasingly being recognized, and abundant evidence has confirmed the intimate relationship between WMI and poor functional outcomes. Although certain pathological factors, including inflammatory injury, oxidative stress and neuro-excitotoxicity, are responsible for ICH-induced WMI, the definite underlying mechanisms need to be further elucidated. However, both inflammatory and oxidative signaling pathways are involved in WMI partially due to the iron overload after ICH. Therefore, treatments targeting iron may be quite promising. From this perspective, the results of the ICH Deferoxamine (iDEF) trial, which will be revealed next year, are highly anticipated. Regarding WM restoration, regulating factors promoting/inhibiting axonal regeneration and remyelination should be defined under the background of ICH. Oligodendrocyte proliferation and differentiation play a central role in remyelination which is involved in the complex crosstalk among oligodendrocytes, astrocytes, microglia and cerebral endothelial cells. An understanding of the cell-cell interaction mechanisms will likely promote oligodendrogenesis. Regarding WMI diagnosis, novel biochemical and neuroimaging markers are expected to detect to assist in the early detection of WMI and the dynamic monitoring of WMI. Finally, for better clinical translation, future experimental treatment strategy designs should provide protection to both GM and WM by targeting multiple pathological factors in poly-gyrated animals rather than using only rodents, and more *in vivo* studies of WMI in patients are desperately needed.

## Author Contributions

CY and CT conceived and designed the manuscript. All authors contributed to the writing of the manuscript. XH and HL edited the manuscript and all authors reviewed the final manuscript.

## Conflict of Interest Statement

The authors declare that the research was conducted in the absence of any commercial or financial relationships that could be construed as a potential conflict of interest.
